# Monkeypox and Peru´s eternal unpredictability

**DOI:** 10.17843/rpmesp.2022.393.12203

**Published:** 2022-09-30

**Authors:** Lely Solari

**Affiliations:** 1 Instituto Nacional de Salud, Lima, Peru. Instituto Nacional de Salud Lima Peru

Many people wonder why Peru, once again, leads the list of Latin American countries in terms of cases per million during the ongoing monkeypox epidemic ^1^. After the great losses of lives and resources that we experienced during the COVID-19 pandemic, we hoped that the experience would have left us with enough tools to better face emerging epidemic challenges. Regarding this issue, reflections made at the global level perfectly apply to our country ^2^. However, once again, we see Peru leading ominous morbidity rankings. Fortunately for us, the monkeypox virus requires close contact for transmission; therefore, the national burden of disease is not perceived as very relevant. But what would have happened had transmission been more effective? Or what would happen if some serious emerging health problem, for instance, caused by climate change, reaches Peru? Reviewing the causes that lead us to this paralysis in health is an essential exercise.

In this context, it is necessary to reflect about some reactions that often arise in the face of epidemic challenges. The first is to look for those who are responsible for the situation. In the specific case of Monkeypox, there have been several attempts to directly blame the population of men who have sex with other men, a population that has historically been stigmatized and marginalized. Factors related to their sexual practices have been considered the key to the transmission of the virus. Although it is true that the virus spreads mostly through close contact, sexual contact, and that the most affected population is that of homosexual and bisexual men, their proportion in our country is not particularly high, nor does our community´s behavior varies significantly from that of other countries in the region ^3^ ^,^ ^4^. It is true that there are practices associated with this group that causes the virus to spread, but these do not explain why the epidemic has a greater epidemiological impact in Peru. 

Another hypothesis that has been proposed is that there is a greater capability to detect the virus in our country in comparison to other countries in the region. This reasoning stems primarily from the fact that we achieved, during the COVID-19 pandemic, a functional, albeit not perfect, flow of specimens for diagnosis from the sampling points to the regional referral laboratories and through them to the national referral laboratory, ensuring access to molecular diagnosis through the Network of Public Health Laboratories ^5^. For example, more than 600 health facilities nationwide have taken samples for the diagnosis of monkeypox. Likewise, the capabilities developed in genomic sequencing have allowed us to recognize that there were more than twelve different entry points of the virus into the country and that the variant with the greatest dispersion is B.1.6, which probably had a local origin ^6^. 

The second element that leads us to consider a high detection power of the virus in the country is the possibility of people living with HIV/AIDS to opportunely access healthcare thanks to an effective HAART network and being used to frequent clinical research studies on HIV. This network of healthcare and clinical research groups has built trust over the years in the population of men who have sex with men. However, the supposed outstanding effectiveness of the system in identifying cases is not in line with the very high percentage of positivity of the samples seen in recent weeks - greater than 60% - which suggests that many cases are not being diagnosed. This situation has probably caused the spread of the epidemic to almost all the regions of the country and to other population groups, such as heterosexual men, women and even children. Likewise, in recent weeks, we have received the report of cases of patients who attended health facilities in late stages, with severe complications that required surgical management and critical care. All this drives away the explanation of the high number of cases. 

We must consider that the approach to a complex health problem such as monkeypox, and previously COVID-19, leads us to reflect on three topics: infrastructure, evidence for decision-making and behavior and values ​​of the population. In terms of infrastructure, when it comes to the social aspect, there is an urgent need to provide the population with access to safe housing, basic sanitation and formal work, which ensures that people affected by infectious diseases can be properly isolated. In terms of health, still in the infrastructure topic, there is an urgent need to strengthen, and even reconstruct our health system, ensuring real universal coverage and a solid base of primary health care. This is important because at the beginning of the monkeypox epidemic, the vast majority of those affected were people living with HIV/AIDS. Had they had a user-friendly and effective primary care system, with staff using appropriate language to approach the contacts, they could have been diagnosed earlier, when we still had a chance of containing transmission, highly centralized in Lima. However, in order to ask for personal information, you first need to build trust. In order to engage the population, it is necessary to prove that the system can provide care ^8^. A classically mistreated and marginalized population demanded an individualized approach, from trained and specialized professionals to be able to make the first call, to commit the contacts and offer them the necessary resources for their isolation, if necessary. Our system does not have that management capacity. On the contrary, an innovative initiative such as the one described would have been quickly questioned by “the experts,” while the officials that could have decided to fund it would have been immediately investigated and threatened by the control system.

Regarding the topic of evidence and decision-making, the pandemic has exposed a scientific fragility to which we have been blinded for decades, both in the state and in academia and business, but most sadly in the population, which lacks critical thinking. Let us consider the example of research studies, where in a simplified way, the analysis can be descriptive, that is, the most basic approach. Then the next step is to evaluate the association between two variables, including the development of experimental models, and finally we reach a multivariate analytical stage, in which many elements are considered at the same time to explain an outcome. This can be used to understand a health situation or to predict and model what is expected to happen in the future ^7^. In our country, since we do not have conditions to support the scientists in their development, people with advanced analytical capacity inevitably migrate to countries where they have greater possibilities to develop as researchers ^8^. Thus, while in other countries they use artificial intelligence to make public health decisions, in Peru the situational analysis is purely descriptive. We limit ourselves to count cases, and the risk communication we can deliver with descriptive information is very incomplete. Let us hope that, even though the transmission of the monkeypox virus continues, no transmission cycles in animals that perpetuate transmission and make us an endemic country appear.

Finally, regarding the topic of values, it is necessary to examine our objectives as a population. During the COVID-19 pandemic, working together to strengthen the health system and be better prepared for future challenges was, for the first time in history, a priority for all Peruvians. With the return to our chaotic normality, we lack a national action plan to ensure adequate responses to future health challenges as an important goal of society. But this phenomenon of relaxation is not restricted to the health sector, in many other sectors such as education and transportation we perceive the same dynamic of “letting be.” To address this issue, we require a commitment at the highest level in order to organize ourselves and organize the processes, at least at a basic level. We can do it and we proved so at the beginning of the vaccination campaign against COVID-19. It does not require an extraordinary effort or resources, but it does require consensus on the objective, adequate management capacity and use of the evidence, to attain the obligations agreed. Nonetheless, could it be that deep down we want to be organized, or could it be that in Peru we feel rather comfortable with the lack of rules and limits? Many of us consider that our individual decisions are better, smarter, more creative than what the system can offer us: we listen to our intuition and judgment. The problem is that out there are millions of intuitions and criteria keeping us in a chronic chaos from which it is increasingly difficult to exit. Either we stop pretending surprise when we see Peru once again occupying a tragic first place in the face of great floods, epidemics, and other disasters to come, or we consider once and for all the possibility of committing ourselves to providing all Peruvians, with the very basic services, use at least elementary scientific reasoning, and follow essential social rules.
