# Supplementary material for: Monkeypox and Peru´s eternal unpredictability
Source: Rev Peru Med Exp Salud Publica. 2022 Sep 30;39(3):264–6. doi: 10.17843/rpmesp.2022.393.12203 (PMC11397687; doi:10.17843/rpmesp.2022.393.12203)
Supplement: Translation

# Translation: Viruela del mono y la eterna impredictibilidad en el Perú

**Authors:** Lely Solari

**Affiliations:** 1 Instituto Nacional de Salud, Lima, Perú

Muchas personas se preguntan por qué el Perú, una vez más, está en el primer lugar de casos por millón en Latinoamérica de la epidemia de turno, que en estos meses es la viruela del mono ^1^. Después de las grandes pérdidas de vidas y recursos que tuvimos con la pandemia de la COVID-19, esperábamos que la experiencia nos hubiera dejado herramientas suficientes para enfrentar mejor los retos epidémicos emergentes. En ese sentido, las reflexiones realizadas a nivel global aplican muy bien a nuestro país ^2^. Sin embargo, una vez más, vemos al Perú liderando ominosos ránquines de morbilidad. Afortunadamente, el virus de la viruela del mono requiere un contacto cercano para su transmisión, por lo que la carga de enfermedad a nivel nacional no se percibe como muy relevante, pero ¿qué pasaría si la transmisión hubiese sido más efectiva, o si alguna emergencia sanitaria grave causada por el cambio climático llegara al Perú? Repasar las causas que nos llevan a esta parálisis en salud es un ejercicio necesario.

En este contexto, es necesario comentar algunas reacciones que existen ante los retos epidémicos. La primera es buscar a los responsables (culpables) de la situación. En el caso particular de este virus, hubo varios intentos por responsabilizar directamente a la población de hombres que tienen sexo con otros hombres, una población históricamente estigmatizada y marginalizada. Factores relacionados con sus prácticas sexuales se han considerado la clave de la diseminación del germen. Si bien es cierto, el virus se propaga a través del contacto cercano, en particular el sexual, y que la población más afectada es la de hombres homo y bisexuales, su proporción en nuestro país no es particularmente elevada, ni nuestra comunidad tiene una conducta sexual que varía significativamente de la de otros países de la región ^3^ ^,^ ^4^. Es cierto que hay conductas asociadas con esta población que hacen que el virus se propague, pero estas no necesariamente explican por qué en el Perú la epidemia tiene una mayor repercusión epidemiológica.

Otra hipótesis que ha sido ensayada es que en nuestro país hay una mayor capacidad de detección del virus con relación a otros de la región. Este razonamiento nace del hecho, en primer lugar, de haber conseguido durante la pandemia de la COVID-19 un flujo funcional, aunque no perfecto, de muestras desde los puntos de obtención de muestra, a los laboratorios de referencia regional y a través de ellos al laboratorio de referencia nacional, asegurando el acceso al diagnóstico molecular a través de la Red Molecular de Laboratorios en Salud Pública ^5^. Por ejemplo, más de 600 establecimientos de salud a nivel nacional han sido capaces de tomar muestras para el diagnóstico de viruela del mono. Asimismo, las capacidades desarrolladas en secuenciación genómica nos han permitido reconocer que hubo más de doce diferentes entradas del virus al país y que la variante de mayor dispersión es la B.1.6 que, probablemente, haya tenido un origen local ^6^.

El segundo elemento que lleva a suponer una mayor detección del virus en el país es el hábito de las personas viviendo con VIH/SIDA de acudir rápidamente a recibir atención gracias a una Red TARGA efectiva y contar con servicios articulados de investigación clínica en VIH que, a través de los años, han forjado confianza en la población de hombres que tienen sexo con hombres. No obstante, esta supuesta sobresaliente efectividad del sistema en captar los casos no concuerda con el altísimo porcentaje de positividad de las muestras que se han visto en las últimas semanas -mayor al 60%- que sugiere que muchos casos deben estar ocurriendo sin ser diagnosticados. Esto posiblemente ha generado la dispersión de la epidemia a casi todas las regiones del país y a otros grupos poblacionales como los hombres heterosexuales, mujeres e incluso niños. Asimismo, en las últimas semanas se ha reportado casos de pacientes que acudieron, de manera tardía, a los establecimientos de salud, con complicaciones severas que requirieron manejo quirúrgico y cuidados críticos, lo que aleja la explicación del alto porcentaje de casos diagnósticados.

La aproximación a un problema sanitario complejo, como el de la viruela del mono, y en su momento la COVID-19, nos lleva a reflexionar sobre tres ejes: el de la infraestructura, el de la evidencia para toma de decisiones y el eje de la conducta y los valores de la población. En lo referente a infraestructura, en el aspecto social nunca se podrá resaltar de manera suficiente la necesidad de brindar a la población acceso a viviendas seguras, saneamiento básico y trabajo formal, lo cual asegura que las personas afectadas por enfermedades infecciosas se puedan aislar durante el tiempo necesario. En el eje estructural del aspecto sanitario, es una necesidad urgente fortalecer, e incluso refundar nuestro sistema de salud, asegurando una cobertura universal real y una base sólida de atención primaria de salud. Al inicio de la epidemia de viruela del mono, la gran mayoría de afectados eran personas viviendo con VIH/SIDA. De haber tenido un sistema de atención primaria amigable y efectivo, con personal utilizando un lenguaje adecuado para abordar a los contactos, estos hubieran podido ser captados tempranamente cuando existía una posibilidad de contener la transmisión, la cual estaba altamente centralizada en Lima. Sin embargo, para solicitar información, en primer lugar, se necesita generar confianza. Para comprometer a la población, se necesita probar que el sistema es capaz de cuidar de ella ^8^. Una población clásicamente maltratada y marginalizada necesitaba un abordaje individualizado, de personas entrenadas y especializadas para poder hacer esa primera llamada, comprometer a los contactos y ofrecerles los recursos necesarios para su aislamiento, de ser el caso. Nuestro sistema todavía no tiene esa capacidad de gestión, por el contrario, una iniciativa innovadora como la descrita, hubiera sido rápidamente cuestionada por «los expertos», en tanto que aquellos funcionarios que decidieran financiarlas, serían inmediatamente investigados y amenazados por el sistema de control, dado que hubiera sido necesario. 

En el eje de la evidencia y toma de decisiones, la pandemia ha dejado al descubierto una precariedad científica ante la cual nos hemos cegado durante décadas, tanto en el estado como en la academia y la empresa, y tristemente en la población, la cual carece de pensamiento crítico. Tomemos el ejemplo de los estudios de investigación, donde de manera simplificada, el análisis puede ser descriptivo, es decir, la aproximación más básica, luego se realiza la evaluación el uso de mecanismos no convencionales en el estado para ejecutarla de la asociación entre dos variables, incluyendo el desarrollo de modelos experimentales, y por último viene una etapa analítica multivariada, en la cual se toman en cuenta muchos elementos al mismo tiempo para explicar un desenlace. Esto puede servir para entender una situación de salud o para predecir y modelar lo que se espera que ocurra en el futuro ^7^. En nuestro país, al no existir las condiciones que apoyen a los científicos en su desarrollo, personal con capacidad analítica avanzada inexorablemente migra a países donde existen mayores posibilidades de desarrollarse como investigadores ^8^. Así, mientras que en otros países utilizan inteligencia artificial para tomar decisiones en salud pública, en el Perú el análisis situacional que podemos hacer es descriptivo. Contamos casos, y la comunicación de riesgo a la población que se puede realizar con información descriptiva es muy incompleta. Esperemos tener la suerte de que, a pesar de que la transmisión del virus de la viruela del mono continúe, no ocurran ciclos de transmisión en animales que perpetúen los casos y nos conviertan en un país endémico. 

Por último, en lo referente al eje de los valores es necesario examinar nuestros objetivos como población. Durante la pandemia de la COVID-19, trabajar juntos para fortalecer el sistema de salud y estar mejor preparados ante retos futuros fue, por primera vez en la historia, una prioridad para todos los peruanos. Con la vuelta a nuestra caótica normalidad, carecemos de un plan de acción nacional para asegurar adecuadas respuestas a futuros retos sanitarios como meta importante de la sociedad en conjunto. Esto no solo pasa por el sector salud, en muchos sectores como educación y transporte se percibe la misma dinámica de «dejar pasar». Para abordar este tema, se requiere un compromiso del más alto nivel, un compromiso de organizarnos y ordenar, aunque sea un poco, los procesos. Somos capaces de hacerlo y lo comprobamos en los inicios de la campaña de vacunación contra la COVID-19. No se requiere de un esfuerzo ni recursos exorbitantes, pero sí se necesita consenso en el objetivo, adecuada capacidad de gestión y uso de la evidencia, para cumplir los compromisos asumidos. Pero ¿será que en el fondo queremos estar organizados, o será que los peruanos nos sentimos más bien cómodos con la falta de reglas y límites? Muchos consideramos que nuestras decisiones individuales son mejores, más inteligentes, más creativas que lo que nos puede ofrecer el sistema: le hacemos caso a la intuición y al criterio. El problema es que hay millones de intuiciones y criterios manteniéndonos en un caos crónico del cual es cada vez más difícil salir. O ante las grandes inundaciones, epidemias y otros desastres por venir, dejamos de fingir sorpresa cuando veamos al Perú ocupando nuevamente un trágico primer lugar, o consideramos de una vez la posibilidad de comprometernos a proveer a todos los peruanos, aunque sea los servicios básicos, usar un razonamiento científico por lo menos elemental y seguir las reglas sociales esenciales.
